# Influence of Freeze Drying and Spray Drying on the Physical and Chemical Properties of Powders from *Cistus creticus* L. Extract

**DOI:** 10.3390/foods14050849

**Published:** 2025-03-01

**Authors:** Alicja Kucharska-Guzik, Łukasz Guzik, Anna Charzyńska, Anna Michalska-Ciechanowska

**Affiliations:** 1 Laboratorium Analiz Chemicznych Spark-Lab Sp. z o.o., Aleja Zwycięstwa 96/98, 81-451 Gdynia, Poland; lukasz.guzik@spark-lab.pl (Ł.G.); anna.buczkowska@spark-lab.pl (A.C.); 2 Department of Fruit, Vegetable and Plant Nutraceutical Technology, Faculty of Biotechnology and Food Science, Wrocław University of Environmental and Life Sciences, ul. Chełmońskiego 37, 51-630 Wrocław, Poland

**Keywords:** plant extracts, inulin, maltodextrin, *Cistus creticus* L. powder, polyphenols

## Abstract

This study aimed to evaluate the feasibility of producing and characterizing *Cistus creticus* L. powders obtained through spray drying and freeze drying using maltodextrin and inulin as carriers. Quantitative and qualitative analysis of polyphenols by high-performance liquid chromatography with diode-array detection (HPLC-DAD) and high-performance liquid chromatography coupled with tandem mass spectrometry (HPLC-MS/MS) identified key bioactive compounds, including punicalagin isomers and their galloyl esters, as well as flavonoids (myricetin-3-galactoside, myricetin-3-rhamnoside, quercetin-3-galactoside, and tiliroside). Phenolics in powders produced by both drying techniques ranged from 73.2 mg to 78.5 mg per g of dry matter. Inulin proved to be as effective as maltodextrin in spray drying, offering a promising alternative for plant-based powder formulation. Antioxidant capacity measured by Trolox equivalent antioxidant capacity assay with 2,2′-azino-bis(3-ethylbenzothiazoline-6-sulfonic acid) (TEAC ABTS) and ferric reducing antioxidant power (FRAP) assay indicated that spray-dried powders with inulin exhibited antioxidant properties comparable to those with maltodextrin. The results demonstrated that *Cistus creticus* L. powders obtained with inulin can serve as valuable sources of bioactive compounds with potential health benefits similar to those obtained with maltodextrin. Moreover, from a technological perspective, inulin proved to be an equally efficient carrier in terms of production-process parameters such as moisture content and water activity, making it a viable alternative to maltodextrin in plant-based powder formulations.

## 1. Introduction

*Cistus creticus* L. is a perennial plant native to Western Asia, Europe, and the Mediterranean basin. It is a rich source of polyphenols, which are recognized for their anti-inflammatory, anti-allergenic, antimicrobial, and antioxidant properties [1,2]. These properties are attributed to the presence and content of, among others, phenolic acids and flavonoids, including rosmarinic acid, quercetin, catechin, and gallic acid [2].

Drying is commonly applied to prolong the availability of plants, including herbs, in the market. Various studies have investigated the effects of different drying techniques on the antioxidant properties of selected parts of *Cistus creticus* L. As an example, Stępień et al. [3] evaluated the influence of convective, vacuum microwave, and combined drying techniques on the total polyphenol content and antioxidant capacity of leaves. It was indicated that combined drying was the most efficient method for preserving bioactivity while minimizing processing time and energy consumption. Similarly, Matłok et al. [4] demonstrated that combined drying retained higher polyphenol content compared with freeze drying or convective drying. Additionally, aromatic compounds such as eugenol, thymol, and carvacrol, which exhibit antiseptic activity, were better preserved in leaves subjected to combined drying.

The development of functional food ingredients or dietary supplements often requires the conversion of plant materials into more stable product-powders, which are usually produced from liquid forms, i.e., extracts obtained by freeze drying or spray drying. This approach is particularly suitable for obtaining soluble products, as the liquid form ensures the effective incorporation of bioactive compounds and carrier agents, resulting in homogenous and easily dissolvable powders. From an economic and sustainability perspective, spray drying is advantageous due to its scalability and lower energy requirements compared with freeze drying [5]. This makes it a more feasible option for industrial applications where cost-effectiveness is critical. In the case of *Cistus creticus* L., Ammendola et al. [6] investigated spray drying of extracts with carboxymethylchitosan as a carrier, highlighting its potential for microencapsulation of bioactive compounds. The addition of carriers prior to drying may affect the processing parameters and the final quality of products. Previous research suggests that carriers not only improve plant powder stability, but can also influence the phenolic composition and, as a result, the biological activity of powders [7]. In addition, carriers can enhance powder properties, such as flowability and encapsulation efficiency, while protecting against oxidation and moisture during storage [8,9]. What is more, spray drying has been shown to effectively reduce moisture content and water activity (*a_w_*), producing stable, microbiologically safe plant powder. The application of these drying techniques directly influences the bioavailability of phenolic compounds.

Maltodextrin is one of the most frequently used carriers in spray drying and freeze drying due to its high solubility, neutral taste, and ability to form a protective matrix around bioactive compounds. It is widely applied in food and pharmaceutical formulations to improve powder stability, enhance encapsulation efficiency, and reduce hygroscopicity. Studies have shown that maltodextrin effectively prevents oxidative degradation of polyphenols and flavonoids. However, alternative carriers such as inulin have gained increasing interest due to their additional health benefits [7]. Recently, inulin, as a prebiotic carrier, gained attention for powder production [10]. Inulin is a naturally occurring polysaccharide classified as a fructan and primarily composed of fructose units linked by β-(2→1) glycosidic bonds. It is commonly found in plants such as chicory (*Cichorium intybus*) and Jerusalem artichoke (*Helianthus tuberosus*). Due to its gel-forming ability, inulin is widely used in the food industry as a stabilizer, fat replacer, and dietary fiber [7]. Additionally, as a prebiotic, inulin may enhance bioavailability by interacting with gut microbiota, offering a dual function as both a stabilizer and a health-promoting agent [11].

Although some studies have explored the relationship between drying techniques and the antioxidant capacity of *Cistus creticus* L., there is limited research on the use of carriers, i.e., inulin and maltodextrin for freeze drying and spray drying of herb extracts. Previous studies have mainly examined the impact of drying techniques on total polyphenol content and antioxidant capacity The potential influence of different carrier types on the stability, phenolic composition, and functionality of the resulting powders remain insufficiently investigated. Additionally, the comparative analysis of inulin and maltodextrin as carriers in the drying process of *Cistus creticus* L. extracts is lacking, particularly in terms of their effects on powder properties and phenolic retention. The study hypothesized that the drying technique (freeze drying or spray drying) and carrier type (maltodextrin or inulin) influence the antioxidant properties, phenolic compound content, and overall quality of the powders derived from *Cistus creticus* L. extract. Our research aimed to fill this gap in knowledge by evaluating the changes in physical attributes and chemical properties, including phenolic content and antioxidant capacity, of powders produced using these two drying techniques. Chemical properties were investigated also by using high-performance liquid chromatography coupled with tandem mass spectrometry (HPLC-MS/MS) to assess the potential use of these powders as functional food ingredients or dietary supplements and to verify the possibility of replacing commonly used maltodextrin with functional carrier, i.e., inulin.

## 2. Materials and Methods

### 2.1. Reagents

Gallic acid, chlorogenic acid, and Folin–Ciocalteu phenol reagent were obtained from Sigma-Aldrich (Darmstadt, Germany), sodium carbonate was purchased from Avantor Chemicals (Gliwice, Poland), 2,2′-Azino-bis(3-ethylbenzothiazoline-6-sulfonic acid) and potassium persulfate were obtained from Merck (Darmstadt, Germany). Acetonitrile hypergrade for HPLC-MS/MS from Merck (Darmstadt, Germany) and formic acid HPLC-MS/MS grade from VWR (Darmstadt, Germany) were used. Deionized water (HLP5, Hydrolab, Straszyn, Polska) was used for sample preparation and chromatography. The cut off conductivity of deionized water was 4.3 µS/cm.

### 2.2. Materials

Approx. 10 kg of *Cistus creticus* L. (dried, crushed herb; moisture content: approx. 7% ± 0.31) was purchased from a HerbaNordPol-Gdańsk Sp. z o.o. (Gdańsk, Poland) in August 2022 and was stored at ambient temperature. Maltodextrin (Pepees S.A., Łomża, Poland) and inulin (Beneo-Orafti, Oreye, Belgium) (food grade) were used as carriers for freeze drying and spray drying.

### 2.3. Methods

#### 2.3.1. Extraction

For the extraction, 25 g of dried *Cistus creticus* L. was combined with 1 L of distilled water at 100 °C in a sealed container and kept at this temperature in a water bath for 1 h, stirring occasionally with a glass rod. Subsequently, the extract was filtered through a nylon filter and the carriers, i.e., maltodextrin and inulin at the concentration of 5% (*w/w*), were added (experimentally established). The carriers were directly dissolved in the extract under continuous stirring to ensure solubilization. The liquid feeds were subjected to drying.

#### 2.3.2. Drying

The liquid feeds were freeze dried (FD) using a FreeZone 18 L freeze dryer (Labconco Inc., Kansas City, MO, USA) under reduced pressure of 65 Pa with the temperature of the drying chamber at −60 °C, and heating plate at 25 °C for 24 h. The process was done in duplicate (*n* = 2). Spray drying (SD) was carried out using a B-290 mini spray dryer (Buchi, Flawil, Switzerland) at inlet air temperature of 170 °C, and outlet air temperature of 102 ± 1 °C. Extract-carrier solutions had a temperature of 23 °C at the entrance to the spray dryer, the feed flow rate was 4 mL/min and air flow was 37 m^3^/h. The process was done in duplicate (*n* = 2).

#### 2.3.3. Physical Properties of Powders

##### Moisture Content (*Mc*)

The *Mc* was determined in duplicate (*n* = 2) using the vacuum-oven method described by Figiel [12]. Samples were vacuum dried at 80 °C for 24 h, and the results are reported as a percentage of wet basis.

##### Water Activity (*a_w_*)

The *a_w_* was determined at 25 °C in duplicate (*n* = 2) using a dew point water activity meter (Model 4TE, AQUA LAB, Pullman, WA, USA).

##### Color, Browning Index (BI), and Yellowness Index (YI)

The color of the powders was assessed (*n* = 4) using a Minolta Chroma Meter CR-400 colorimeter (Minolta Co., Ltd., Osaka, Japan) based on the CIE L*a*b* color space. The BI was determined using Equation (1), proposed by Palou [13] and Rhim [14]. The YI was determined using Equation (2), proposed by Rhim [14].

(1) $$BI=\frac{100\times (x-0.31)}{0.172}$$

where

$$x=\frac{a+1.75L}{5.645L+a-3.012b}$$

(2) $$YI=\frac{142.86\times b}{L}$$

##### Particle Size Analysis

The particle size distribution was performed using a Malvern Mastersizer 2000 (Malvern Panalytical, Malvern, UK) liquid analyzer using isopropanol as the dispersing liquid. The dispersant was selected to eliminate the phenomenon of sample dissolution. The device was configured by introducing appropriate instrument constants: absorption coefficient 0.1; refractive index at the glass/isopropanol interface 1.39. The measurements were done in duplicate (*n* = 2).

#### 2.3.4. Chemical Properties

##### Qualitative and Quantitative Analysis of Powders from *Cistus creticus* L. by HPLC-MS/MS Technique

The qualitative analysis of polyphenolic compounds was conducted using a liquid chromatography system equipped with a DAD detector (Shimadzu, Kyoto, Japan) and a Phenomenex Luna Omega column (Phenomenex, Torrance, CA, USA) (C_18_, 1.6 µm 100 × 2.1 mm; 100 Å). The chromatographic separation was achieved via gradient elution with a mobile phase consisting of phase A (1% formic acid in water, *v/v*) and phase B (1% formic acid in acetonitrile, *v/v*) at a flow rate of 0.3 mL/min. The elution was performed using a step gradient as follows: 0–15 min, 95% A and 5% B; 15–55 min, 80% A and 20% B; 55–62 min, 60% A and 40% B; and 62.5–70 min, 95% A and 5% B. The injection volume was set to 5 µL, with the column temperature maintained at 30 °C and the autosampler temperature at 15 °C. Analytical wavelengths (190–700 nm) for the DAD detector were set according to the compound class being analyzed. The diluent was a 95:5 (*v*/*v*) mixture of phase A and phase B. Samples were prepared by dissolving 1 g of powder in 100 mL of diluent, sonicating for 3 min, centrifuging at 3226 rcf for 5 min at 25 °C, and analyzing 1.5 mL of the supernatant. Standard solutions of chlorogenic acid were prepared for calibration, with working solutions diluted to 10 µg/mL for quantitative analysis, dissolved in the diluent. Data acquisition and analysis were performed using a specified software system, and results are expressed as mg/g of dry matter (DM). Each sample was analyzed in duplicate (*n* = 2). The identification of compounds was carried out on one selected sample of *Cistus creticus* L. dried by spray drying with inulin. Identification of compounds in the other extracts was done by comparing peak retention times against a reference sample.

To evaluate the qualitative profile of polyphenols, the LC-MS/MS technique (Shimadzu, Kyoto, Japan; LCMS-8040 with LabSolutions software, ver. 5.75) was employed, using the same mobile phases, column, and gradient conditions as described above. The MS detector parameters for the method included SCAN mode within the range of 100–1500 *m/z*, both ESI+ and ESI- ionization modes (4.5 kV ionization energy), with nitrogen (purity 99.0% (*v/v*), produced in nitrogen generator) used as the nebulizing gas (flow rate: 3 L/min) and drying gas (flow rate: 8 L/min). The interface temperature was 250 °C, and the desolvation line was 400 °C. Analyses were carried out at a scanning speed of 1034 u/sec. To determine the identity of the compounds, the M+ [*m/z*] and M- [*m/z*] parameter data were compared with available literature data [4]. Moreover, product ion scans PRIS(+) and PRIS(-) were performed. The selected precursor ions were fragmented using different collision energies: 15, 35, 45, and 50 eV and argon as collision gas (purity 99.9999% (*v/v*)). These data were compared with data from scientific literature [15,16,17].

##### Reducing Potential

The extraction process followed the method outlined by Michalska-Ciechanowska et al. [18]. The reducing potential of the powdered aqueous extract of *Cistus creticus* L. with added carrier was analyzed using the Folin–Ciocalteu method, as described by Gao et al. [19] and modified by Horszwald and Andlauer [20]. In this method, the sample reacts with Folin–Ciocalteu reagent under alkaline conditions, resulting in a blue complex, which is quantified spectrophotometrically at λ = 750 nm. The analysis was performed with a Synergy H1 spectrophotometer (BioTek Instruments Inc., Santa Clara, CA, USA). The results (*n* = 2) were reported as grams of gallic acid equivalents (GAEs) per 100 g DM.

##### Antioxidant Capacity In Vitro

The antioxidant capacity of the powders was evaluated using the TEAC ABTS and FRAP assays according to the methods described by Re et al. [18] and Benzie and Strain [19]. The TEAC ABTS assay is based on the ability of antioxidants to scavenge the ABTS^+^ radical cation, while the FRAP assay measures the reduction of Fe(III)-TPTZ (Fe(III)-2,4,6-Tris(2-pyridyl)-s-triazine) complex to Fe(II)-TPTZ in an acidic environment. The measurements were conducted in duplicate (*n* = 2), and the results are reported as mmol Trolox equivalent (TE) per 100 g of DM.

#### 2.3.5. Statistical Analysis

The data were statistically evaluated using STATISTICA 13 software (StatSoft, Tulsa, OK, USA). The mean values and standard deviations were analyzed through ANOVA. To identify statistically significant differences between groups, Tukey’s post hoc test (Tukey HSD) was applied with a significance level of *p* < 0.05.

## 3. Results and Discussion

### 3.1. Extraction of Cistus creticus *L.*

**Figure 1:**
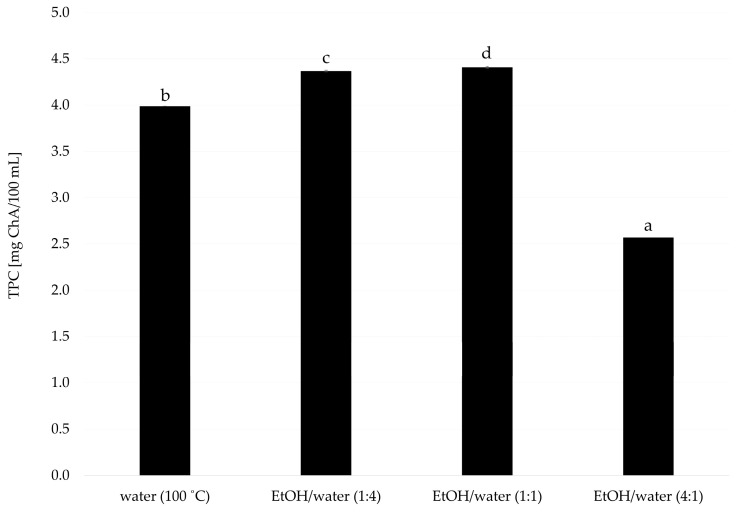
Comparison of extractants of dry leaves of *Cistus creticus* L. ChA—chlorogenic acid; ^a–d^—values followed by the same letter are not statistically significantly different (*p* < 0.05) (ANOVA, Tukey’s HSD test).

To determine the suitable solvent for extract preparation, various solvents were evaluated (Figure 1). Among the solvents and their mixtures, the highest reducing potentials measured by the method with Folin reagent were indicated for samples gained with the addition of the ethanol–water mixture (1:1; *v/v*), demonstrating the effectiveness of balanced polarity in dissolving both polar and nonpolar compounds. Water extraction at 100 °C resulted in extracts with a reducing potential of approximately 4 mg/100 mL. The reducing potential was similar for both 1:1 and 1:4 *(v/v*) mixtures, highlighting the role of high temperature in breaking cellular structures and releasing polyphenols.

Although ethanol–water mixtures showed the highest efficiency, water at 100 °C was chosen for further research due to its environmentally friendly nature, safety, and simplicity in industrial applications. This followed previous studies indicating that water, particularly at elevated temperatures, is an effective and sustainable solvent for polyphenol extraction from plant materials [21,22]. This approach ensured a “green solvent” choice, supporting both efficiency and sustainability in the production process.

### 3.2. Physical Properties of Powders

#### 3.2.1. Moisture Content

The *Mc* of samples with maltodextrin and inulin was relatively low (below 4.5%) (Table 1), as both drying techniques effectively reduced moisture content through water evaporation resulting in fine, dry powders suitable for storage [23]. Both drying techniques resulted in similar moisture content, with no statistically significant differences found regardless of the carrier used. These findings suggest that the samples were stable and safe for storage [24,25,26].

#### 3.2.2. Water Activity

The *a_w_* is a key factor in determination of foods stability and shelf life, reflecting water availability for chemical reactions and microbial growth. Lower *a_w_* generally improves microbial stability and extends shelf life, especially in powdered products [24]. In this study, all samples had *a_w_* values below 0.15 indicating that the products were biochemically and microbiologically stable [27]. Overall, spray drying resulted in samples with slightly lower *a_w_* values compared with freeze-dried ones; however, no statistically significant differences were detected (Table 1). Among the spray-dried samples, powders containing maltodextrin displayed slightly higher *a_w_* values than those produced with inulin. Previous research suggested that the type of carrier and the presence of components in the liquid feed (in this case, the herbal extract) could influence interactions between these components, thereby affecting the properties of the final product [15]. This may result from rapid water evaporation during spray drying, but both products are likely to be stable during storage [23]. Freeze-dried samples showed a reversed trend, with maltodextrin having slightly lower *a_w_* than powders with inulin.

In summary, both drying techniques resulted in powders with a relatively low water activity, and carriers did not differentiate the samples from this parameter’s point of view. Thus, inulin can be successfully applied for production of powdered products based on *Cistus creticus* L. extracts.

#### 3.2.3. Color, Browning Index (BI), and Yellowness Index (YI)

The color parameters (CIE L*, a*, and b*), BI, and YI are key factors of the visual characteristics of powders that may be influenced by drying techniques and carrier types. Freeze-dried products generally were lighter (a higher coordinate L* values), with inulin producing the brightest samples (Table 1). Based on the results presented, spray-dried powders were darker than freeze-dried products, with maltodextrin yielding slightly higher values than inulin showing that the drying technique and carrier applied may affect the color of powders [28,29].

The a* parameter values exhibited statistically significant differences among the samples, influenced by both the drying technique and the type of carrier used. Freeze-dried samples with inulin exhibited lower a* values, indicating a more greenish hue, whereas samples with maltodextrin displayed a slight reddish tint. For spray-dried samples, powders with inulin had higher a* values compared with those with maltodextrin, indicating a shift toward a redder hue. In contrast, spray-dried powders with maltodextrin exhibited lower a* values, suggesting a slightly greener tone [30]. In the case of coordinate b*, the drying technique had stronger effect only in the case of maltodextrin.

Where BI was concerned, it was shown that inulin-added products had higher values of BI when spray drying was applied for their dehydration, whereas addition of maltodextrin before freeze drying resulted in higher BI. Thus, the type of carrier and parameters used for drying may have altered the BI of the products obtained [25]. Similarly, yellowness index was consistently higher for maltodextrin, especially in freeze-dried products [29].

In summary, freeze drying led to the obtainment of lighter samples favoring inulin for applications requiring minimal color changes. Spray drying intensified color, with maltodextrin enhancing yellowness and browning; it is therefore suitable for products where these traits are desirable. Drying techniques and carriers altered the visual properties of powders (Supplementary File S1).

#### 3.2.4. Particle Size Distribution

**Figure 2:**
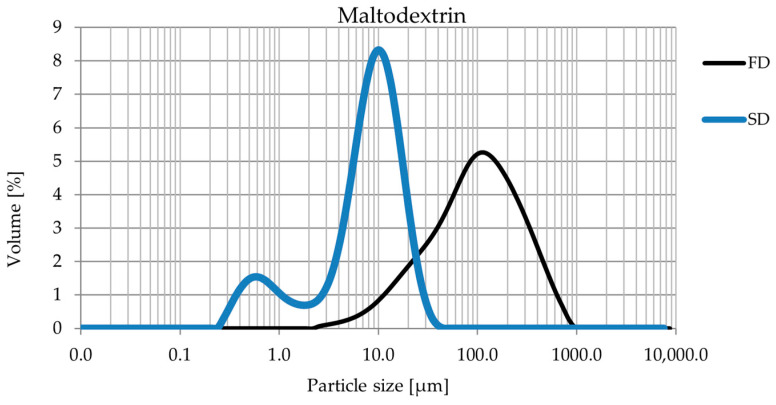
Comparison of the particle size distribution of *Cistus criticus* L. extract obtained by freeze drying (FD) with maltodextrin and spray drying (SD) with maltodextrin. The graph shows the volume share [%] as a function of particle size [µm].

**Figure 3:**
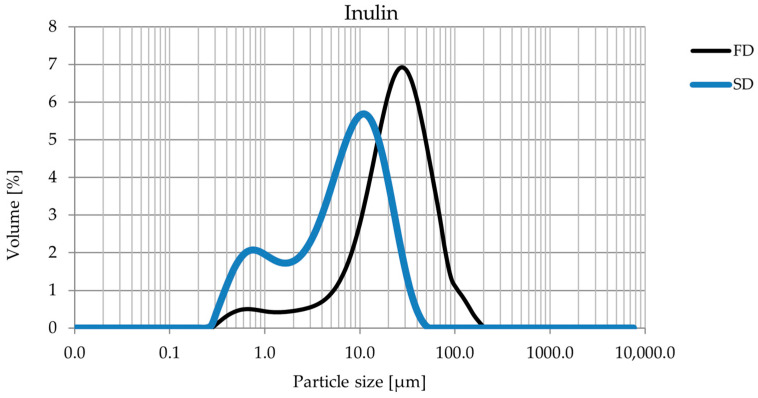
Comparison of the particle size distribution of *Cistus criticus* L. extract obtained by freeze drying (FD) with inulin and spray drying (SD) with inulin. The graph shows the volume share [%] as a function of particle size [µm].

The analysis of particle size distribution in samples subjected to SD and FD revealed significant differences caused by the applied techniques. The SD samples had a more homogeneous particle size distribution, with a significantly lower proportion of both the finest and largest fractions (Figure 2 and Figure 3). Freeze drying, on the other hand, generated particles with a larger size.

Additionally, the results indicated that the type of carrier used also influenced the final particle size, particularly in freeze-dried samples (Table 2). Powders obtained with maltodextrin exhibited significantly larger particle sizes compared with those produced with inulin. This difference can be attributed to the distinct physicochemical properties of the carriers. Maltodextrin forms a more cohesive matrix during freezing, leading to the development of larger structures that remain after sublimation. In contrast, inulin, due to its different water-binding capacity, results in a more fragmented and less compact matrix, yielding smaller particles after freeze drying [31].

The differences obtained can be attributed to the specificity of the processes. Spray drying, as reported in the literature, typically produces smaller and more uniform particles due to the rapid evaporation of the water, which limits particle agglomeration. This technique is often preferred for applications requiring precise and stable particle size distribution, such as pharmaceutical formulations and functional foods [26,32].

Freeze drying, on the other hand, tends to result in a wider particle size range, as the process involves the sublimation of water at low temperature and reduced pressure, which can lead to increased variability in particle sizes [33]. The carrier type plays a crucial role in this process, as its ability to interact with water and form a solid matrix during freezing directly affects the final particle structure. While maltodextrin contributes to the formation of larger and more cohesive structures, inulin appears to limit excessive particle growth, resulting in a finer powder with a narrower distribution range.

The results indicated that the choice of drying technique has a significant impact on the particle size distribution and thus on the potential use of the final product. Freeze drying, generating larger particles with a broader distribution range (Table 2), may be preferable for products requiring reduced cohesion and improved flow properties, while spray drying, providing greater uniformity, may be more suitable for applications where precise particle size control is required.

In summary, drying significantly affects particle size distribution and potential product applications. Spray drying resulted in smaller and more uniform particles, which were commonly associated with improved dispersion and stability in formulations. Freeze drying produced larger particles with a wider distribution range, which could benefit applications where a lower bulk density or free-flowing texture is desirable. Larger powder particles (greater than 100 µm) may have a better channeling parameter, especially with the encapsulation process. Finer powders (e.g., less than 100 µm) have a higher specific surface area, which favors van der Waals and electrostatic forces, increasing adhesion between particles. Fine powders can form bridges and aggregates, leading to clumping and impeded flow.

### 3.3. Chemical Properties

#### 3.3.1. Qualitative Determination of Polyphenols in *Cistus creticus* L. Powders

**Figure 4:**
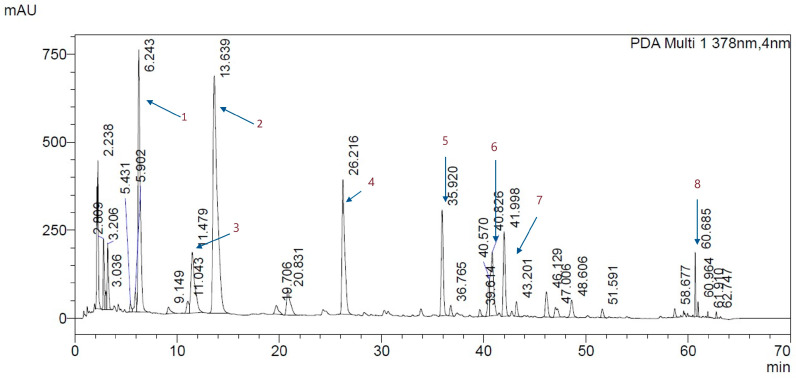
Chromatogram of *Cistus creticus* L. powder analyzed by UHPLC-MS/MS. 1—punicalagin isomer; 2—galloyl-punicalagin isomer; 3—punicalagin isomer; 4—galloyl-punicalagin isomer; 5—myricetin-3-galactoside; 6—myricetin-3-rhamnoside; 7—quercetin-3-galactoside; 8—tiliroside.

The qualitative analysis of polyphenols in the spray-dried extract of *Cistus creticus* L. with inulin, conducted using HPLC-MS/MS, indicated a diverse profile of ellagitannins and flavonoids (Figure 4). Triple quadrupole mass spectrometry analysis in positive and negative ionization modes allowed for precise identification of key compounds based on retention times (RTs) and molecular masses (*m/z* values).

Punicalagin isomers were identified as major ellagitannins in the analyzed powders (Table 3). These compounds are characteristic of *Cistus* species and are known for their relatively strong antioxidant properties due to their multiple hydroxyl groups [34]. Their high abundance suggests that these constituents may play a significant role in the bioactivity of the extract.

In the analyzed samples, gallated derivatives of punicalagin were also detected. The identification of these derivatives highlights the complexity of ellagitannins in *Cistus* powders. Another component identified was the glycosylated flavonol, i.e., myricetin-3-galactoside. Myricetin derivatives are known for their antioxidant, anti-inflammatory, and potential neuroprotective effects [35]. Myricetin-3-rhamnoside, which is a rhamnose-conjugated derivative of myricetin, enhances the stability and solubility of the flavonol, potentially contributing to the extract’s bioactivity [36]. Quercetin-3-galactoside, also known as hyperoside, is a widely distributed flavonoid with potent antioxidant activity and cardioprotective effects [37].

Tiliroside, a kaempferol glycoside, was identified as a prominent compound. Known for its anti-inflammatory and antimicrobial properties, this flavonoid glycoside further enriched the bioactive profile of the powders [38].

The identified polyphenolic compounds, particularly punicalagin isomers, gallated ellagitannins, and flavonoid glycosides, may contribute to the bioactivity of the extract through multiple mechanisms. Their antioxidant properties allow them to neutralize reactive oxygen species (ROS), thereby reducing oxidative stress [34,37]. Additionally, their anti-inflammatory effects may stem from the inhibition of pro-inflammatory mediators [37], while antimicrobial properties could be linked to interactions with bacterial cell membranes [38]. These combined effects suggest a potential role in the therapeutic applications of *Cistus creticus* L. extract.

#### 3.3.2. Quantitative Determination of Main Polyphenols of *Cistus creticus* L. by HPLC-DAD

The quantitative analysis of polyphenols in *Cistus creticus* L. powders was conducted using the HPLC-DAD technique. The study evaluated the content of selected bioactive compounds in samples subjected to FD and SD, with maltodextrin and inulin applied as carriers (Table 4).

The analysis identified and quantified compounds such as gallocatechin-(4α-8)-gallocatechin, and punicalagin isomers and their galloyl esters, along with key flavonoids, including myricetin-3-galactoside, myricetin-3-rhamnoside, quercetin-3-galactoside, and tiliroside.

For the majority of compounds, including gallocatechin-(4α-8)-gallocatechin, myricetin-3-galactoside, and myricetin-3-rhamnoside, no statistically significant differences in content were observed between the samples. However, slight variations were noted in the concentrations of punicalagin isomers and their galloyl derivatives, which may reflect the differing stability of these compounds under varying drying conditions. Additionally, the content of quercetin-3-galactoside and tiliroside exhibited some fluctuations depending on the type of carrier and the applied drying technique.

The total polyphenol content remained comparable across all samples, indicating that both freeze drying and spray drying were effective in preserving the overall phenolic profile of *Cistus creticus* L. powders.

#### 3.3.3. Antioxidant Capacity In Vitro

The results demonstrated that the drying technique and carrier type significantly influenced the antioxidant properties of powders, including their reducing potential and antioxidant properties (TEAC ABTS and FRAP).

Products dried with inulin via freeze drying exhibited the highest overall reducing potential and antioxidant capacity measured by TEAC ABTS and FRAP, outperforming all other combinations of drying techniques and carriers (Table 5). In contrast, maltodextrin showed significantly lower retention of antioxidant compounds under freeze-drying conditions, possibly due to its limited protective capabilities during sublimation (Supplementary File S2).

Spray drying, despite involving relatively high temperatures, showed favorable results when inulin was used as the carrier. Powders produced by spray drying with inulin had a higher reduction potential of 10.7% and antioxidant capacity compared with those with maltodextrin. This indicates that inulin provides similar or even slightly higher protective effect against thermal degradation, making it a suitable carrier for applications requiring efficient antioxidant retention even under high-temperature drying. In previous studies, it was shown that the protective effect of inulin on antioxidant capacity during spray drying can be attributed to its structural and physicochemical properties. Inulin, as a polysaccharide, forms a matrix that encapsulates bioactive compounds, reducing their direct exposure to high temperatures and oxidative stress. This encapsulation effect limits the degradation of polyphenols and other antioxidants by creating a physical barrier against thermal and oxidative degradation [7,39].

These results highlight that both spray drying and freeze drying with inulin are effective approaches for preserving the antioxidant potential of powders. The choice of method should depend on the specific application, with spray drying offering a faster, cost-effective process and freeze drying maximizing bioactive compound retention. This aligns with research emphasizing the role of drying technologies and carrier materials in determining the final product quality [32,40].

## 4. Conclusions

The conducted study confirmed the possibility of producing *Cistus creticus* L. powders using both spray drying and freeze drying methods with maltodextrin and inulin as carriers having moisture content below 4.5% and water activity below 0.15.

Quantitative and qualitative analysis of polyphenols using HPLC-DAD and HPLC-MS/MS revealed the presence of key bioactive compounds, such as punicalagin isomers, their galloyl esters, and flavonoids (myricetin-3-galactoside, myricetin-3-rhamnoside, quercetin-3-galactoside, and tiliroside). Both SD and FD effectively preserved the overall phenolic profile of the powders. No reports were found in the literature regarding the qualitative and quantitative analysis of polyphenols in *Cistus creticus* L. powders with the addition of inulin and maltodextrin, highlighting the innovative nature of this research.

The results of antioxidant activity analysis (TEAC ABTS and FRAP) confirmed that spray powders with inulin exhibited a comparable level of antioxidant capacity to samples with maltodextrin.

The results showed that inulin is an equally effective carrier as maltodextrin in spray drying, making it an attractive alternative for the production of plant-based powders. Such an approach offers new possibilities in the creation of functional foods and pharma products. These findings might help in developing innovative strategies for enhancing the bioavailability and stability of phenolic compounds while ensuring scalability and economic feasibility for industrial applications.

## Figures and Tables

**Table 1:** Selected physical properties of *Cistus creticus* L. powders.

Drying Technique	Carrier Type	Moisture Content [%]	Water Activity [-]	Color			BI	YI
				L*	a*	b*		
FD	Maltodextrin	4.36 ± 0.08 ^a^	0.107 ± 0.004 ^a^	74.47 ± 1.43 ^ab^	0.18 ± 0.02 ^ab^	32.97 ± 0.5 ^b^	55.97 ± 1.6 ^b^	63.27 ± 1.38 ^b^
	Inulin	3.42 ± 0.49 ^a^	0.114 ± 0.002 ^a^	77.96 ± 0.8 ^b^	−0.86 ± 0.07 ^a^	28.25 ± 0.29 ^ab^	42.33 ± 0.43 ^a^	51.76 ± 0.38 ^a^
SD	Maltodextrin	3.42 ± 0.49 ^a^	0.105 ± 0.017 ^a^	69.14 ± 4.37 ^ab^	−0.26 ± 0.11 ^ab^	26.67 ± 1.36 ^a^	46.43 ± 0.71 ^a^	55.12 ± 0.63 ^a^
	Inulin	3.57 ± 0.01 ^a^	0.096 ± 0.005 ^a^	66.31 ± 3.21 ^a^	0.88 ± 0.65 ^b^	28.74 ± 2.22 ^ab^	55.22 ± 2.87 ^b^	61.87 ± 1.94 ^b^

FD—freeze drying; SD—spray drying; BI—browning index; YI—yellowness index; ^a,b^—values followed by the same letter in a column are not statistically significantly different (*p* < 0.05) (Tukey’s HSD test).

**Table 2:** Particle size distribution (d10, d50, and d90) of powders obtained using spray drying (SD) and freeze drying (FD) with maltodextrin and inulin as carriers.

Drying Technique	Carrier Type	d10 [mm]	d50 [mm]	d90 [mm]
FD	Maltodextrin	16.63 ^c^	79.43 ^c^	275.42 ^c^
	Inulin	5.44 ^b^	23.37 ^b^	60.87 ^b^
SD	Maltodextrin	0.83 ^a^	7.59 ^a^	17.38 ^a^
	Inulin	0.72 ^a^	6.61 ^a^	19.95 ^a^

FD—freeze drying; SD—spray drying; d10—particle size below which 10% of the sample’s total volume was found (represents the fine fraction of the sample); d50 (median particle size)—particle size at which 50% of the sample’s total volume was smaller and 50% was larger (the median of the particle size distribution); d90—particle size below which 90% of the sample’s total volume was found (indicates the coarser fraction of the sample). ^a,b,c^—values followed by the same letter in a column are not statistically significantly different (*p* < 0.05) (Tukey’s HSD test).

**Table 3:** Identification of polyphenol compounds in *Cistus creticus* L. powder using its spectral characteristics, positive and negative ions in HPLC-MS/MS [17].

Peak	RT (min)	*m/z* (Positive)	*m/z* (Negative)	*m/z* PRIS Ions (+/- Mode)	Collision Energy [eV]	Compound Name	Class
1	6.243	-	1083	601; 781; 575 (-)	45	Punicalagin isomer ^1^	Ellagitannin
2	11.479	-	1251	1207; 1083 (-)	35	Punicalagin-gallate isomer ^1^	Ellagitannin
3	13.639	-	1083	601; 781; 575; 721; 299 (-)	50	Punicalagin isomer ^2^	Ellagitannin
4	26.216	-	1251	1207; 1083 (-)	45	Punicalagin-gallate isomer ^2^	Ellagitannin
5	35.92	481	479	316; 271; 287; 151; 214 (-)	45	Myricetin-3-galactoside	Flavonoid (flavonol)
6	40.826	465	463	316; 271; 287 (-)	35	Myricetin-3-rhamnoside	Flavonoid (flavonol)
7	41.998	465	463	300; 271; 255 (+)	35	Quercetin-3-galactoside	Flavonoid (flavonol)
8	60.685	595	593	285; 307; 447 (-)	15	Tiliroside	Flavonoid glycoside

RT-retention time. Isomers 1 and 2 refer to different structural variants.

**Table 4:** Determination of polyphenolic content in *Cistus creticus* L. powders using HPLC-DAD.

Compound	FD		SD	
	Maltodextrin	Inulin	Maltodextrin	Inulin
	[mg/g DM]			
Gallocatechin-(4α-8)-gallocatechin	10.8 ± 0.9 ^a^	10.4 ± 0.3 ^a^	11.4 ± 0.3 ^a^	9.9 ± 0.6 ^a^
Punicalagin isomer ^1^	13.2 ± 0.1 ^a^	12.2 ± 0.5 ^a^	13.3 ± 0.5 ^a^	12.2 ± 0.1 ^a^
Punicalagin-gallate isomer ^1^	11.3 ± 0.1 ^a^	10.9 ± 0.1 ^a^	11.6 ± 0.4 ^a^	10.7 ± 0.3 ^a^
Punicalagin isomer ^2^	22.0 ± 0.2 ^a^	20.5 ± 0.2 ^a^	21.7 ± 0.1 ^a^	25.8 ± 4.7 ^a^
Punicalagin-gallate isomer ^2^	11.5 ± 0.02 ^ab^	11.0 ± 0.4 ^ab^	12.0 ± 0.3 ^b^	10.3 ± 0.4 ^a^
Myricetin-3-galactoside	1.8 ± 0.01 ^a^	1.7 ± 0.01 ^a^	1.8 ± 0.01 ^a^	1.9 ± 0.2 ^a^
Myricetin 3-rhamnoside	4.3 ± 0.1 ^a^	4.1 ± 0.02 ^a^	4.2 ± 0.1 ^a^	3.7 ± 0.4 ^a^
Quercetin-3-galactoside	1.8 ± 0.01 ^a^	1.8 ± 0.01 ^a^	1.9 ± 0.1 ^a^	2.3 ± 0.1 ^b^
Tiliroside	0.6 ± 0.01 ^ab^	0.5 ± 0.01 ^a^	0.6 ± 0.01 ^ab^	0.7 ± 0.1 ^b^
TOTAL	77.2 ^a^	73.2 ^a^	78.5 ^a^	77.5 ^a^

All values are based on a calibration curve with a phenolic standard—chlorogenic acid; FD—freeze drying; SD—spray drying; DM—dry matter; Isomers 1 and 2 refer to different structural variants; ^a,b^—values followed by the same letter in a column are not statistically significantly different (*p* < 0.05) (Tukey’s HSD test).

**Table 5:** Antioxidant capacity of *Cistus creticus* L. powders.

Drying Technique	Carrier Type	Reducing Potential [g GAE/100 g DM]	TEAC ABTS [mmol Trolox/100 g DM]	FRAP [mmol Trolox/100 g DM]
FD	Maltodextrin	2.93 ± 0.3 ^a^	29.31 ± 0.56 ^a^	19.52 ± 0.53 ^a^
	Inulin	5.12 ± 0.33 ^b^	42.36 ± 2.15 ^b^	29.3 ± 1.23 ^b^
SD	Maltodextrin	4.58 ± 0.69 ^ab^	36.07 ± 2.13 ^ab^	25.18 ± 2.48 ^ab^
	Inulin	5.07 ± 0.13 ^b^	39.88 ±1.00 ^b^	27.86 ± 0.82 ^b^

FD—freeze drying; SD—spray drying; DM—dry matter; ^a,b^—values followed by the same letter in a column are not statistically significantly different (*p* < 0.05) (Tukey’s HSD test).

## Data Availability

The original contributions presented in this study are included in the article/Supplementary Materials. Further inquiries can be directed to the corresponding authors.

## Supplementary Materials

The following supporting information can be downloaded at: https://www.mdpi.com/article/10.3390/foods14050849/s1, Figure S1. Heatmap illustrating color parameters (*L**, *a**, *b**), Browning Index (BI), and Yellowness Index (YI) across different drying methods (SD—spray drying; FD—freeze-drying) and carriers (M—maltodextrin; I—inulin); Figure S2. Heatmap showing reducing potential, FRAP, and TEAC ABTS for different drying methods (SD—spray drying; FD—freeze drying) and carriers (M—maltodextrin; I—inulin).
