# Supplementary material for: Influence of Freeze Drying and Spray Drying on the Physical and Chemical Properties of Powders from Cistus creticus L. Extract
Source: Foods. 2025 Mar 1;14(5):849. doi: 10.3390/foods14050849 (PMC11898460; doi:10.3390/foods14050849)
Supplement: Supplementary file 1 [file foods-14-00849-s001.zip › foods-3453277-supplementary.pdf]

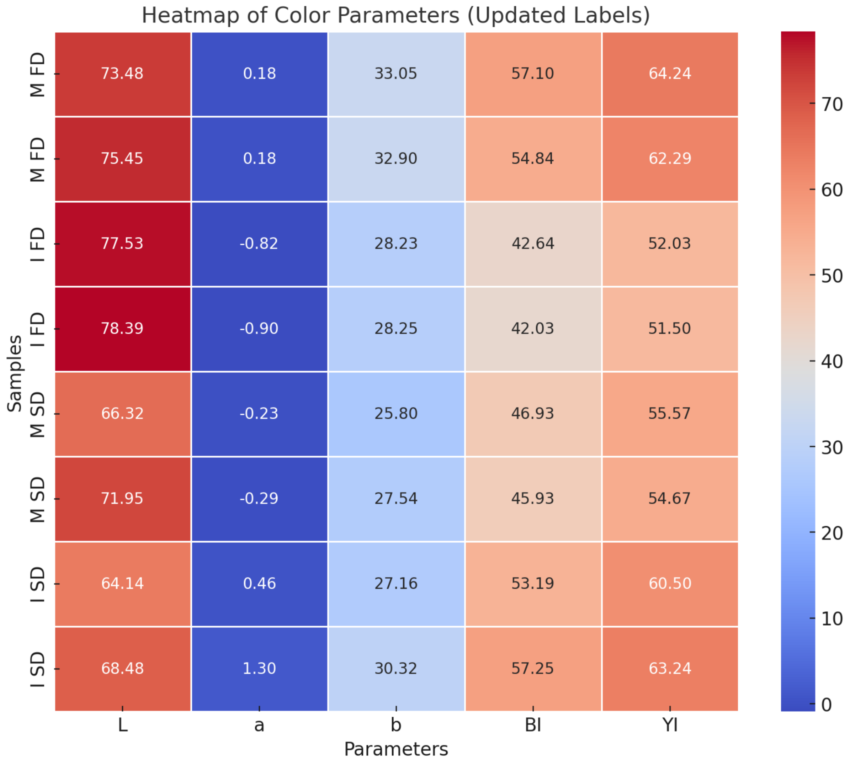

S1. Heatmap illustrating color parameters (L\?a\, b\), Browning Index (BI), and Yellowness Index (YI) across different drying methods (SD – spray drying; FD – freeze-drying) and carriers (M – maltodextrin; I – inulin).

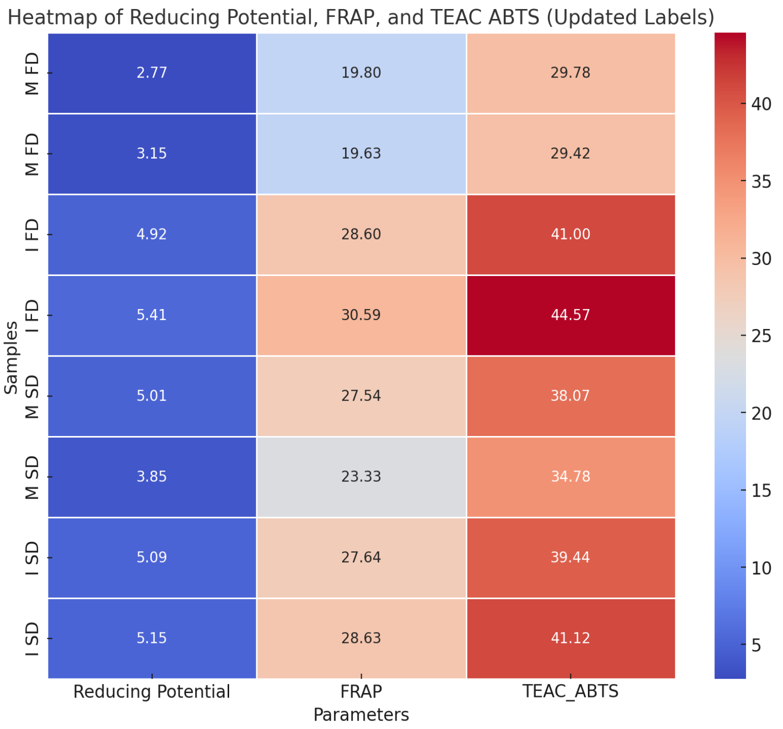

S2. Heatmap showing reducing potential, FRAP, and TEAC ABTS for different drying methods (SD-spray drying; FD-freeze drying) and carriers (M - maltodextrin; I - inulin).
